# Assessment of parental mosaicism rates in neurodevelopmental disorders caused by apparent de novo pathogenic variants using deep sequencing

**DOI:** 10.1038/s41598-024-53358-9

**Published:** 2024-03-04

**Authors:** François Lecoquierre, Kévin Cassinari, Nathalie Drouot, Angèle May, Steeve Fourneaux, Francoise Charbonnier, Celine Derambure, Sophie Coutant, Pascale Saugier-Veber, Alexander Hoischen, Camille Charbonnier, Gaël Nicolas

**Affiliations:** 1 Univ Rouen Normandie, Inserm U1245 and CHU Rouen, Department of Genetics and reference center for developmental disorders, F-76000 Rouen, France; 2 Centre de Ressources Biologiques institutionnel du CHU de Rouen – Biothèque filière génétique, Rouen, France; 3 Department of Human Genetics, Radboud University Medical Center, 6525 GA Nijmegen, The Netherlands

**Keywords:** Genomics, Medical genetics, Mutation, Neurodevelopmental disorders, Sequencing

## Abstract

While de novo variants (DNV) are overall at low risk of recurrence in subsequent pregnancies, a subset is at high risk due to parental mosaicism. Accurately identifying cases of parental mosaicism is therefore important for genetic counseling in clinical care. Some studies have investigated the rate of parental mosaics, but most were either limited by the sensitivity of the techniques (i.e. exome or genome sequencing), or focused on specific types of disease such as epileptic syndromes. This study aimed to determine the proportion of parental mosaicism among the DNV causing neurodevelopmental disorders (NDDs) in a series not enriched in epilepsy syndromes. We collected 189 patients with NDD-associated DNV. We applied a smMIP enrichment method and sequenced parental blood DNA samples to an average depth of 7000x. Power simulation indicated that mosaicism with an allelic fraction of 0.5% would have been detected for 87% of positions with 90% power. We observed seven parental mosaic variants (3.7% of families), of which four (2.1% of families) had an allelic fraction of less than 1%. In total, our study identifies a relatively low proportion of parental mosaicism in NDD-associated DNVs and raises the question of a biological mechanism behind the higher rates of parental mosaicism detected in other studies, particularly those focusing on epileptic syndromes.

## Introduction

De novo variants (DNVs) reflect the genomes’ propensity for change across generations. DNVs are both a driving force of evolution and an important source of diseases. In humans, they represent a major genetic cause of developmental disorders^1,2^. Mutation events are considered to occur in one cell and consist in a change in the genome that is not corrected by the cell’s DNA repair systems. While DNVs can happen in the zygote, such a timing is not the most likely to happen, and most DNVs are hypothesized to occur as a mosaic, either pre-zygotically in parental cells, or post-zygotically in the child^3^. Notably, a large proportion of de novo single nucleotide variants (SNVs) in humans have been suggested to happen following a mutational event in the father’s spermatogonial germ cell. This explains why (i) most DNVs occur on the paternal haplotype (approximately 80% of phased variants^4^) and (ii) the number of DNVs per individuals is under strong influence of the father’s age at conception, as variants accumulate in dividing spermatogonial cells over time during adult men’s life^5^. As only one spermatogonial cell among millions is affected, this subtype of DNV events, termed sperm mosaicism type IIA^3^, is not at risk of multiple transmission to the offspring. Conversely, variants occurring earlier in parental development and affecting multiple daughter germinal cells do have the potential to be transmitted to multiple children. In such parental mosaicisms, variants occur either before primordial germ cell specification (PGCS, type IIIA), or after PGCS (type IIIB). Because of their early onset during embryo development, type IIIA variants are usually observed across multiple tissues of the individuals, notably within the blood cells, and are at high risk of transmission to the offspring. In a medical setting, after the identification of a pathogenic DNV in a child, detecting type III mosaicism may therefore be relevant for genetic counselling given the higher risk of recurrence in a future pregnancy^6^. In addition, because of parental biological sample availability, it appears much more likely to detect type IIIA than type IIIB DNVs, which require the analysis of germinal cells.

Several studies aimed to assess the prevalence of parental mosaicism in presumed DNVs^7^. Some of them used large exome datasets of child-parents trios, obtained from blood samples, and with sequencing depths of coverage adapted to the detection of germline variants. Applying adapted bioinformatics procedures, from 0.3 to 3.4% of DNVs were found to be parental mosaics^8–12^. Specific protocols have also been used to detect parental blood mosaicism using more sensitive techniques on smaller cohorts, including amplicon-based targeted NGS^6,13–15^, digital-droplet PCR (ddPCR)^16,17^ or single molecule molecular inversion probe (smMIP)-based targeted sequencing^18,19^. These various protocols influenced the prevalence of parental mosaicism by increasing the sensitivity to detect parental mosaics, allowing the detection of mosaics with lower allelic ratios. While 5 to 9% of DNVs were found to be a parental mosaic in several studies^13–15,17–19^, those focusing on patients with epilepsy or enriched in such patients showed the highest percentages.

In this study, we sought to establish the prevalence of parental mosaicism for pathogenic variants considered to be de novo in a series a patients with a neurodevelopmental disorder (NDD), not enriched in epilepsy genes. We exploited the smMIPs technique, which uses unique molecular identifiers (UMIs) and deep sequencing, to allow a fine discrimination from background noise. Applying this high sensitivity method to 378 parental samples, we found that 3.7% of DNV were parental mosaics in a cohort not enriched with epilepsy patients.

## Methods

### Patients and samples

All patient and parental samples were referred to the molecular genetics laboratory of the Rouen University Hospital, Rouen, France, in the context of the etiological assessment of a developmental disease in a diagnostic setting. A retrospective series of patients who fulfilled the following two criteria was constituted: (i) presence of a clinically relevant DNV, defined as a probably pathogenic, pathogenic or variant of uncertain significance if it was returned to the clinician, following ACMG-AMP criteria^20^ and (ii) availability of DNA samples from both parents. We included all trios fulfilling the above-mentioned criteria recruited before June 2020, regardless of the mutated gene or the presence or not of a known parental mosaic. This resulted in a recruitment that was representative of the outpatients clinical department consultation of developmental disorder of the Rouen University hospital, from one side (76% of patients), and enriched in patients with Cornelia de Lange syndrome and differential diagnoses, from the other side (24% of patients), with a national recruitment for this latter disorder. Variants had been previously detected on DNA extracted form whole blood samples using simplex or trio exome sequencing, or simplex panel sequencing. In all families, Sanger sequencing was performed in the parents, either as an independent validation of the trio results, or as a segregation analysis for simplex cases. Mendelian concordance had been confirmed for all families in a diagnostic setting before their inclusion in this study. This was achieved either through a custom assay involving 7 polymorphic microsatellites located on autosomes and chromosome X for simplex cases, or by directly assessing Mendelian concordance on SNPs for trio cases. All individuals provided informed consent for genetic analyses and agreed to the use of leftover specimens for research purposes. Parental leftover DNA samples used in this study were obtained from the “Centre de Ressources Biologiques institutionnel du CHU de Rouen” biobank. This study was authorized by the CERDE ethics committee (Rouen University Hospital, Notification N° E2023-33) and performed in accordance with the Declaration of Helsinki.

### smMIP design, sample preparation and sequencing

The technology of smMIPs was used as the enrichment method for targeted deep sequencing in parents. The MIPGEN software^21^ was used to design oligos, with the following parameters: min_capture_size and max_capture_size set to 94 (capture size corresponds to the sum length of ligation arm, extension arm and insert), arm_length_sums set to 40, double_tile_strands_separately set to “on”, to design two smMIPs per variant, one on each genomic strand. Eight consecutive degenerated nucleotides were used as unique molecular identifiers (UMIs). This design resulted in 8 gaps; therefore a total of 189 families could finally be explored. The list of 189 included variants is provided in Table S1 and the MIPGEN design in Table S2. smMIP capture was performed using a protocol described elsewhere^22^. To achieve higher sequencing depth, individuals were divided into 8 pools, so that each individual was not sequenced in all but in only one eighth of the positions (Table S1). Two consecutive preparation runs were performed. In the first run, 200 ng of each DNA sample was used, with a smMIP/haploid genome ratio of 800:1. In the second run, low performing MIPs were rebalanced by using a 20-fold increase compared to non-low performing smMIPs. In this second run, 300 ng of DNA was used, and a 4000:1 smMIP/DNA ratio. Both runs were sequenced on a Nextseq 500 mid output flowcell (130 million clusters) with 2 × 75 bp reads (Rouen genomics facility).

### Bioinformatics procedures

Demultiplexed reads were aligned on GRCh37 using BWA mem. Duplicate reads were flagged using the information from the UMIs and removed using UMI tools. Deduplicated BAM files from run 1 and run 2 were merged using samtools merge. Genotyping was based on the pileup method using samtools pileup from a custom python script (see web resources). Each variant was converted as a chromosome, a position and an alternative motif to look for in the pileup string result. To validate the ability of the pileup method to detect the variants, we first applied it to the exome and panel alignment files of the probands, expected to harbor the variant at a constitutional state. All variants were correctly detected in probands (data not shown). Subsequently, targeted variant detection was performed on smMIP data. Variants of each pool were searched in the two parental samples and 40–48 control samples (i.e. parents of probands with a distinct variant in the pool). Output data consisted in a global genotyping table including ref and alt counts, total count and allelic fraction for parental and control samples.

### Statistics

To detect parental mosaicism at a given position i, the ratio of alternative counts over total counts for each parental sample at position i was compared to the overall ratio of alternative counts over total counts among all control samples of the pool at this position. To account for extremely low allelic ratios among controls, a one-sided Poisson test was used for this comparison. A total of 189 × 2 tests were computed, one for each parent of the 189 patients with DNVs. Therefore a significance level of 0.05/(189 × 2) = 1.3 × 10–4 was required for experiment-wide significance after Bonferroni correction for multiple testing.

We performed a power analysis to identify the allelic fraction lower detection limit for each variant. A detailed grid of possible allelic fractions, from 0.01% to 40%, was put to the test to compute the power curve associated with the one-sided exact Poisson test. For each variant of interest and targeted allele fraction, power was computed for a sample size of the average position sequencing depth among controls at the position, with control allelic fraction at the position as allelic fraction under the null, targeted allele fraction as alternative, and experiment-wide corrected type I error (α = 1.3 × 10^−4^). The github burrm/lolcat package was used for these computations. Finally, for each variant, the smallest allele fraction guaranteeing 90% power was retained as detection limit.

### Digital PCR

Candidate mosaic calls in parents were assessed using digital droplet PCR with standard protocol on a QX200 plateform (Bio-Rad Laboratories). For each variant, a couple of PCR primers was designed, as well as two hydrolysis probes specific of reference (HEX labelled) and alternative (FAM labelled) (Table S3).

## Results

### Description of the series

One hundred and eighty-nine individuals with NDD from 189 families (84 females, 105 males) fulfilled the inclusion criteria and were enrolled for parental targeted deep sequencing after successful design of smMIPs oligonucleotides (Table S1). The majority (144/189) were included for the diagnosis of developmental disorder with or without intellectual disability or autism (exome or targeted gene panel of intellectual disability, n = 44 genes), while 45 were included in a context of a cohesinopathy syndrome (Cornelia de Lange and related disorders, gene panel, n = 22 genes). A total of 186 distinct variants in 107 genes were analysed, consisting in 124 SNVs and 62 indels (range 1–5 nucleotides). One hundred and sixty-one variants were identified through a simplex sequencing assay (panel or exome sequencing, followed by Sanger segregation), while 21 were identified through a trio exome assay. Six variants were known to be mosaic in cases prior to this study. However, these variants were not excluded, ensuring that the distribution of de novo variants aligns in an unbiased manner with the results obtained in clinical routine.

### Sequencing and power analysis

**Figure 1:**
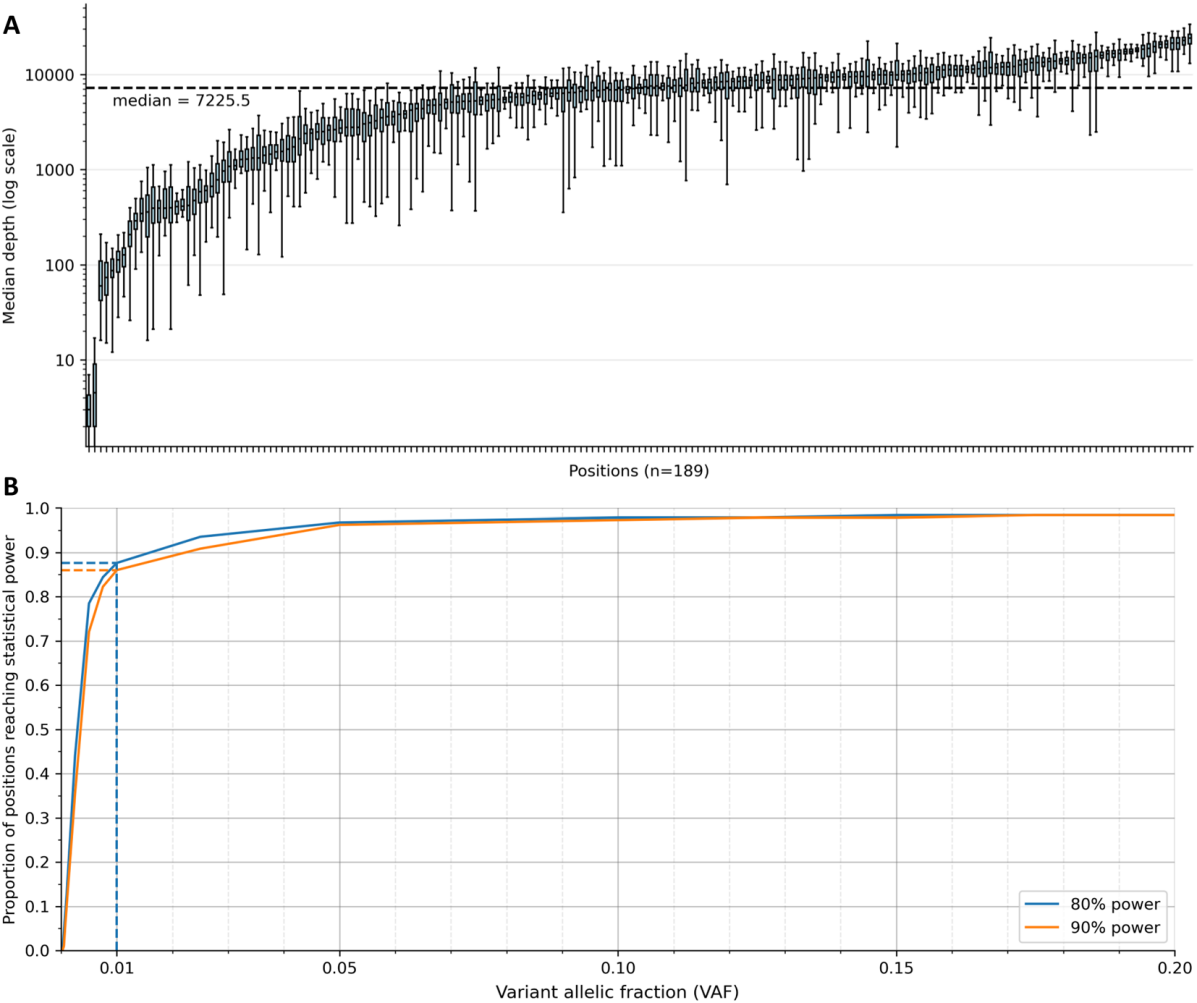
Sequencing depth and power analysis. (**A**) Sequencing depth at the position of each variant, after read deduplication. Each boxplot represents the depth values of both parents plus 46 controls. Logarithmic scale on the y axis. (**B**) Power analysis of the investigated positions. The sequencing noise and depth in controls are integrated to establish the minimum detectable allelic fraction threshold at 80 and 90% power and 5% type-I error after Bonferroni correction for 189 * 2 tests. As an example, a mosaicism above 1% in a parent could theoretically be detected for 87.6% of investigated positions with 80% of power (blue line).

**Figure 2:**
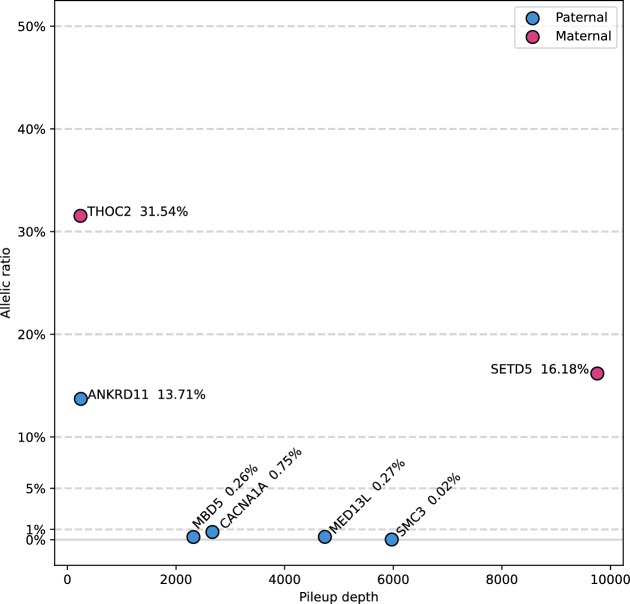
Confirmed parental mosaic variants detected on parental blood samples. Allelic fraction and sequencing depth of the seven confirmed mosaic variants are presented. X-axis depicts the sequencing depth as assessed by the count provided by Samtools Mpileup. Allelic fraction corresponds to alt reads/total within the pileup.

Whole blood DNA samples from both parents, as well as 46 controls, were sequenced at each position. Of note, control samples corresponded to the parents of children harbouring a distinct DNV than the one assessed. A candidate mosaic variant was called if allelic fraction of one parent was significantly higher than that of controls (see “Methods”). A median of 7225 × of coverage was obtained after removal of PCR duplicates (Fig. 1A). The detectability of parental mosaics in the sequencing data differed across the positions studied, due to (i) the difference in sequencing depth between positions (Fig. 1B) and (ii) the short-read sequencing noise that depends on the nucleotide context (Fig. S1). The median allelic fraction at which parental mosaicism would be detected at a 90% sensitivity was 0.5%, with first and third quartiles ranging from 0.25 to 0.75% (Fig. 2). Across all variants under investigation, 87.0% reached more than 90% statistical power at a targeted allelic fraction of 1% and 72.8% at an allelic fraction of 0.5%.

### Identification of parental mosaicism in 3.7% of probands

Seven variants were detected as candidate mosaic variants in father or mother’s samples. Digital droplet PCR validated 6 of them (the seventh was validated by Sanger sequencing), giving a precision of 100% for our statistical approach. Therefore, with the techniques employed, we identified a parental mosaicism in 3.7% of the families investigated (7/189) (Fig. 2). The allelic fraction ranged from 0.02 to 31.5% in the sequencing data, and similar proportions were observed in the digital PCR confirmation (Fig. S2). The variant with the lowest allelic fraction of 0.02% was an indel detected in smMIP data with a single alternative allele-bearing read pair (after duplicates removal) in the father’s sample but none in the controls (Fig. S1), which was statistically significant and could be confirmed by ddPCR at a proportion of 0.03% (Fig. S3). Allelic fraction appeared to be higher in maternal mosaics than in paternal ones, but this effect was not statistically significant.

It should be noted that two of the variations with the highest allelic fractions (in *THOC2* and *ANKRD11*) had already been identified using the Sanger technique during the initial diagnosis prior to this study (these variants were included regardless and analyses were performed in blind of this result). Also, none of the seven detected mosaics were in families sequenced with trio-based exome sequencing, so that we could not interrogate exome sequencing data retrospectively.

As paternal age is strongly associated with variants occurring in spermatogonial cells (non-mosaic variants in our study), we compared the paternal age at conception in the mosaic (n = 7) versus non-mosaic group (n = 168). As expected, we observed that the paternal age at conception tended to be globally lower in the mosaic group, of 4.1 years in average (Fig. S4, 29.6 years vs 33.7 years, *p* = 0.049, Wilcoxon rank sum test). Three out of the 45 DNVs identified by targeted panel in a clinical context of cohesinopathy (6.7%) were found to be a parental mosaicism, while this proportion was as low as 2.8% (4/144, non significant difference, Fisher exact test) within the developmental disorder/ID/autism-associated DNVs (Table S1).

The parent of origin of mosaic variants was biased towards paternal mosaicisms (5/7, 71%), however the number of samples was too small to draw any conclusions. The variant type of parental mosaicisms (6 SNVs and 1 indel) was not significantly different from non-mosaic variants (121 and 61 respectively, *p* = 0.42, Fisher exact test).

## Discussion

To detect parental mosaicism on NDD-causing apparent de novo clinically relevant variants, we sequenced parental and control DNA samples at a median depth of over 7.000x, leading to a high theoretical sensitivity, below 0.5% of allelic fraction in most positions. This strategy resulted in the identification of parental mosaicism in 3.7% of the families (7/189). The detectability of parental mosaics, as the signal-to-noise ratio in parents versus controls, varied across investigated genomic positions. The two main reasons were the disparity in sequencing depth linked to the smMIPs enrichment method (influencing the amount of signal) and the variability of the sequencing noise linked to the nucleotide context. In some sequence contexts, the background noise could be significant. For example, for the NM_032682.5:c.1146 + 1G > A variant, the average allelic ratio in controls was 0.3% and as high as 1% in some controls despite good sequencing depth. At the other extreme, some variants were not associated with any sequencing noise, such as variant NM_005445.3:c.2642_2644del in *SMC3*, which had no alternative reads in the controls and only one alternative read in the father after deduplication of the reads. This signal was detected by our statistical approach and confirmed by ddPCR, with an allelic fraction of 0.03%. The specific nature of this 3-bp deletion in a non-repeated region probably explained the absence of noise, in contrast to substitutions or repeated regions. In summary, we used a high sequencing depth and a large number of controls (n = 46 per variant) to increase the signal-to-noise ratio and improve the detectability of parental mosaics. The use of controls provided a high level of accuracy in detecting candidate mosaics, with a positive predictive value of 100% with our statistical approach, with no effect from the allelic ratio of the mosaicisms.

While our methods theoretically allowed for finer detectability than in most similar studies, we did not identify a larger proportion of parental mosaics. The proportion of 3% is at the lower limit of previous studies on pathogenic DNV^7^. It is uncertain whether pathogenic variants of certain specific diseases could be associated with larger proportions of parental mosaics and what the underlying biological mechanism could be. However, a striking proportion of studies on the parental mosaicism were based on cohorts of patients with epilepsy and particularly Dravet syndrome related to variations in *SCN1A*^13,14,16,18,19^. Most of these studies showed a proportion of parental mosaics of more than 6%, and up to 23%^16^. Post-zygotic mosaics in affected children have also been shown to be frequent in syndromes with epilepsy^23^, but it is not clear if these conditions are enriched in mosaicism compared to other diseases or non-pathogenic variants. Cornelia de Lange syndrome, most frequently caused by *NIPBL* variants, is highly associated with post-zygotic mosaicism in affected cases^24^ and parental mosaicism has also been previously described^25^ in 3.4% to 5.4% families^26^. Our data confirmed the presence of both child (Table S1) and parental mosaicisms in the clinical context of cohesinopathies overall, although only one case of parental mosaicism was a Cornelia de Lange syndrome, with an *SMC3* likely pathogenic variant. Parental mosaicism in cohesinopathies was more prevalent than in developmental disorders/intellectual disability/autism-associated DNVs (6.7% vs 2.8%) but the difference was not statistically significant. Altogether, it is not yet clear whether the DNV associated with specific clinical contexts could be enriched in parental mosaics, and larger-scale studies are needed.

It is interesting to observe that studies based on exome sequencing data in trios of patients with NDDs revealed lower proportions of mosaics than in the current study, but maybe not as much as we could have expected (< 2% compared to ~ 3%), given the much higher sensitivity of our approach compared to exomes with average depth of coverage adapted to germline variants (80–150x)^8–12^. One can speculate that nearly half of the mosaic variants identified here would have been missed by exome sequencing (4 of the 7 variants with an allelic ratio below 1%). If replicated in other studies, our results suggest that applying a much more sensitive technique and similar recruitment results in increased probability of parental mosaic detection, but also that careful examination of sequencing reads in parents remains useful for almost half of the cases.

It is tempting to hypothesize an inverse correlation between the prevalence of parental mosaicism and the proportion of associated mosaicism (i.e. the allelic fraction). Indeed, a strong mosaicism can only derive from a mutational event in a limited number of initial embryonic cells (i.e. at the two or four-cell stage), whereas a low fraction mosaicism can occur in a larger number of cells, increasing its probability. This effect, visible in other studies^27^, could not be observed in our data due to the small number of mosaic variants.

The true proportion of DNV resulting from parental embryonic mosaics (type III) is not known. While it is likely higher than the low estimate of this study, it is improbable that such parental mosaics account for the majority of DNV. Indeed, the major increase in the number of DNV with paternal age and the preponderant proportion of DNV on the paternal haplotype indicate a marked prevalence of mutational events occurring within spermatogonial cells in men of reproductive age (type II sperm mosaicism). This biological effect was present in our data, as the mean paternal age at conception was 4.1 years lower in the mosaic group compared to the non-mosaic group, explained by the type II sperm mosaicism “diluting” the stable mosaic proportion overtime. Given the significantly disparate risks of mutational recurrence between these two groups of variations, their distinction can have a significant impact on the genetic counselling for families. The identification of a parental mosaic indicates a potential risk of mutational recurrence in subsequent pregnancies. An approach based on blood DNA sequencing does not allow for complete sensitivity, as it does not detect mosaicisms that occurred after the specification of the germline (type IIIB mosaicisms), nor an accurate quantification of recurrence risk as the proportion of mosaicism can vary between tissues, such as blood and germinal tissues^27^. While these factors are a limit to the clinical use of our approach, complementary experiments appear promising. First, phasing the DNVs with long-read sequencing techniques could discriminate DNVs occurring on the paternal and maternal haplotype, which diverge in terms of recurrence risk^28^. Second, analysis of the gametes directly, and particularly sperm, which are the most accessible, could allow for a more precise risk quantification^29^. These two approaches have recently been used jointly and efficiently allowed a fine recurrence risk stratification in ~ 70% of 58 families^6^.

In conclusion, using ultra-sensitive techniques, we were able to identify a parental mosaic in 3.7% (7/189) of cases of pathogenic variants causing NDDs. The identification of these mosaics allows the determination of the timing of the mutational event, which corresponds to an embryonic cell of one of the parents during one of its early divisions. To clarify the risk of mutational recurrence and to characterize the cell of appearance of a larger proportion of de novo variations, the study of spermatic DNA could be the subject of subsequent studies.

### Supplementary Information

Supplementary Figures.

Supplementary Tables.

## Data Availability

Variants presented in this study have been submitted to Clinvar: https://www.ncbi.nlm.nih.gov/clinvar/submitters/506968/. All data generated or analysed during this study are included in this published article (and its Supplementary Information files). Pileup script: https://github.com/francois-lecoquierre/smmip_analysis.
