# Supplementary material for: Assessment of parental mosaicism rates in neurodevelopmental disorders caused by apparent de novo pathogenic variants using deep sequencing
Source: Sci Rep. 2024 Mar 4;14:5289. doi: 10.1038/s41598-024-53358-9 (PMC10912112; doi:10.1038/s41598-024-53358-9)
Supplement: Supplementary file 1 — Supplementary Figures. [file 41598_2024_53358_MOESM1_ESM.docx]

**Assessment of parental mosaicism rates in neurodevelopmental disorders caused by apparent *de novo* pathogenic variants using deep sequencing**

Supplementary figures

[Supplementary Figure 1 : Candidate mosaic variants detected in SMMIP data 2](#_Toc156324777)

[Supplementary Figure 2 : Concordance of DDPCR vs SMMIP on allelic balance 3](#_Toc156324778)

[Supplementary Figure 3 : Confirmation of a 0.3% paternal mosaicism in SMC3 by ddPCR 4](#_Toc156324779)

[Supplementary Figure 4: Distribution of paternal age at birth according to mosaic status 5](#_Toc156324780)


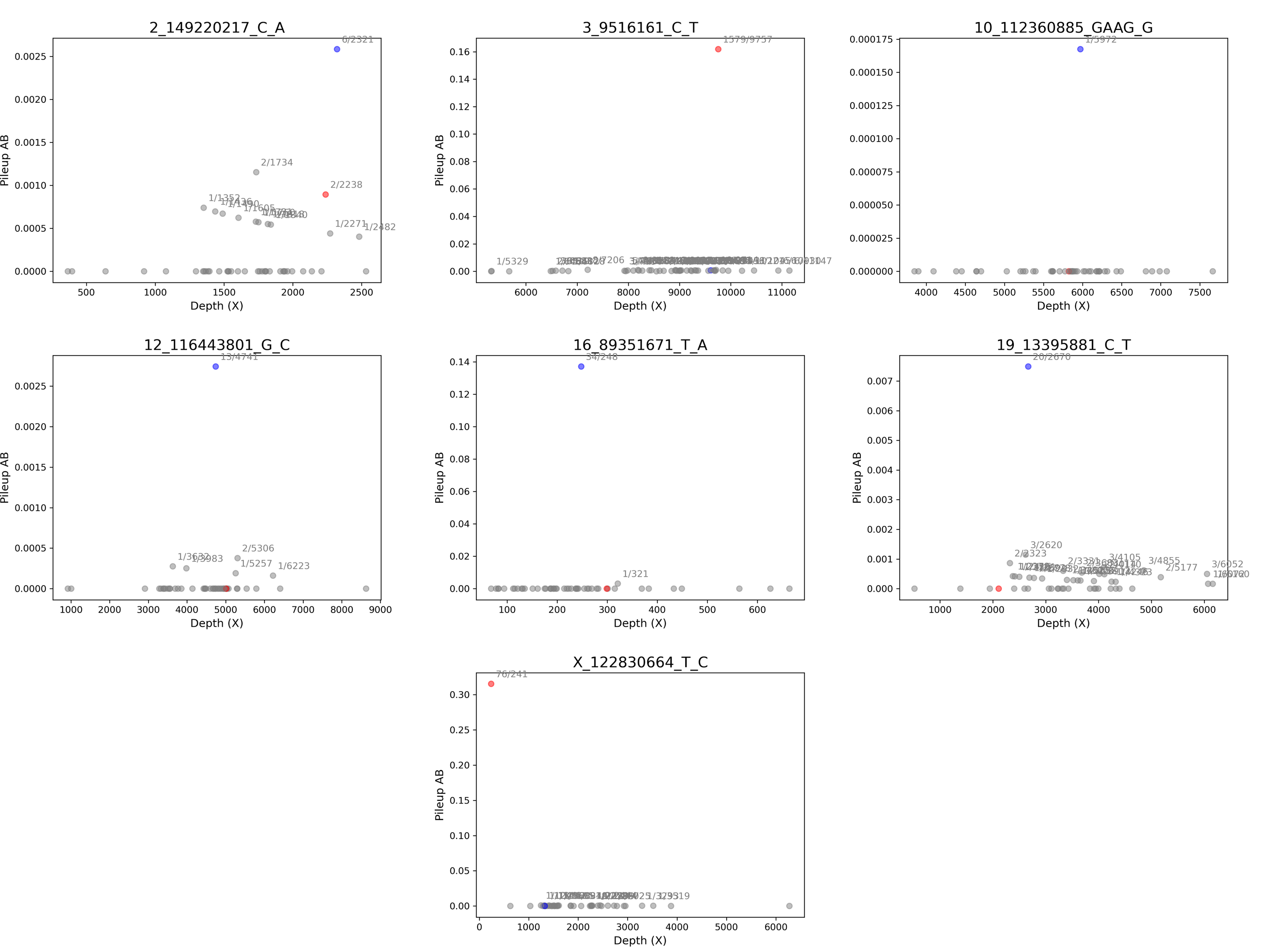


Supplementary Figure 1 : Candidate mosaic variants detected in SMMIP data

Depth is plotted versus allelic balance for the seven candidate mosaic variants detected. Each dot represent a sample, with control samples in grey, mother’s sample in red and father’s sample in blue. All seven variants were subsequently confirmed as true mosaicisms by ddPCR.


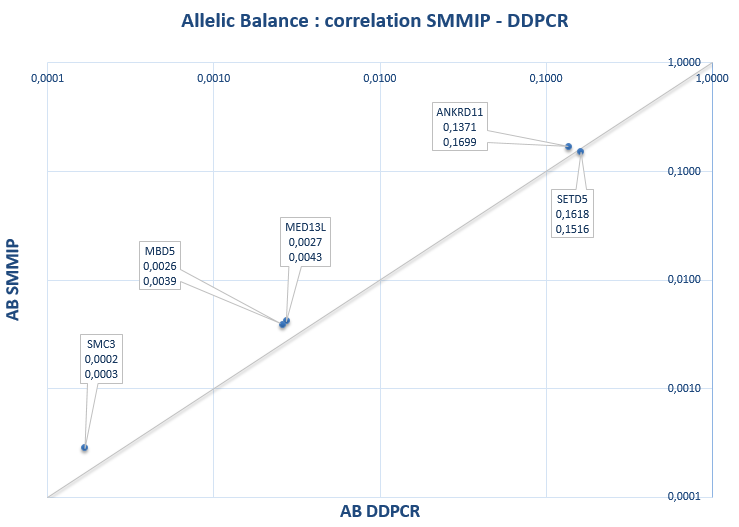


Supplementary Figure 2 : Concordance of DDPCR vs SMMIP on allelic balance

Allelic balance obtained with DDPCR (alt droplets / (alt + ref), see Sup Figure S3) is compared to allelic balance on SMMIP data (alt_reads / total). Logarithmic scale.


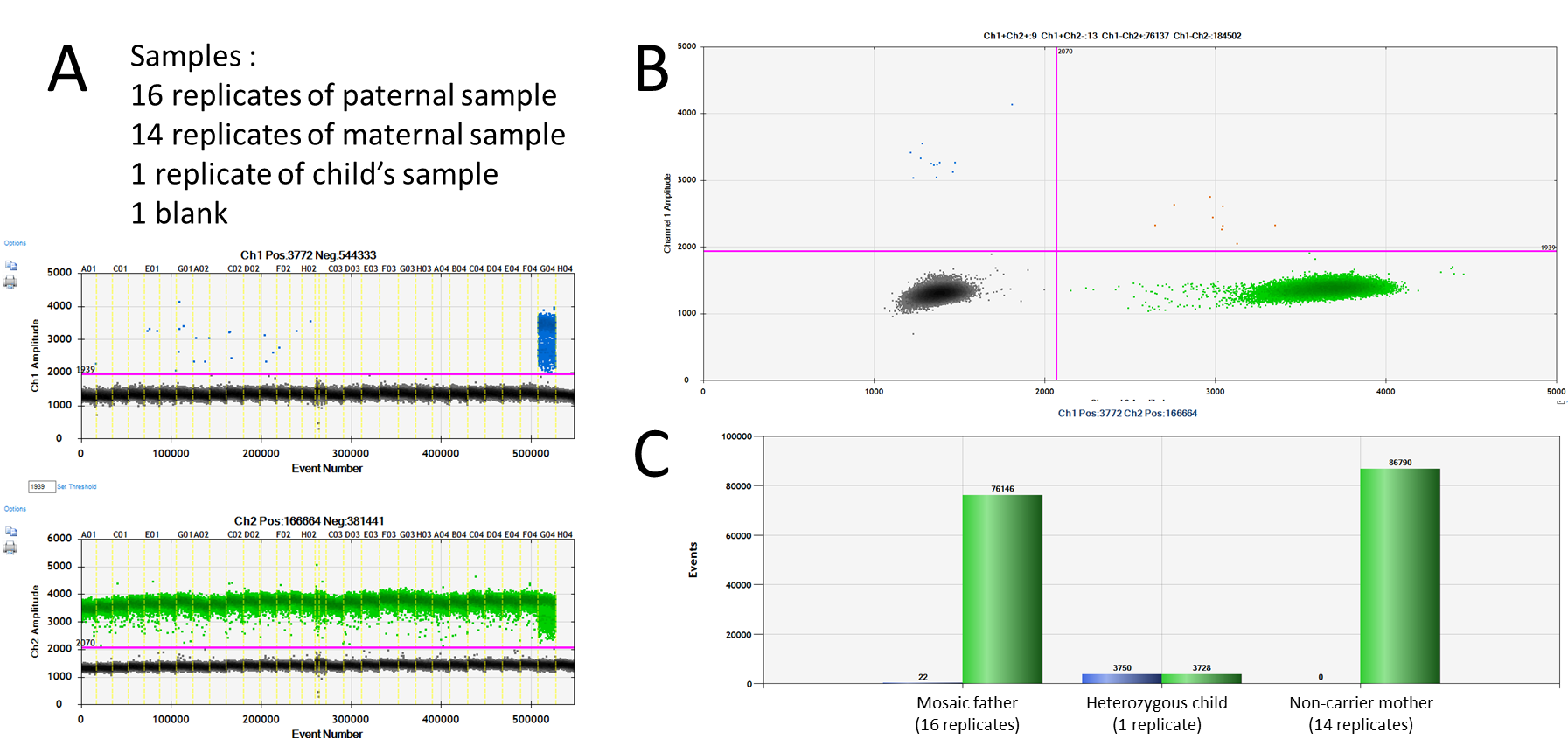


Supplementary Figure 3 : Confirmation of a 0.3% paternal mosaicism in SMC3 by ddPCR

SMC3 variant NM_005445:c.2642_2644del detected as a mosaic candidate by smMIP in paternal sample is confirmed by ddPCR. Green droplets are droplets positive to reference allele-associated dye, and blue droplets are those positive for reference allele-associated dye.

1. 1D plot in father, mother and child samples.
2. 2D plot of the 14 paternal replicates combined. Yellow droplets are positive for both wild type and alternate dyes.
3. Droplet counts. Because of the absence of noise in non-carrier samples (i.e. the maternal sample), these counts allow the calculation of ddPCR allelic fractions using the alt_count/(alt_count + ref_count) formula.


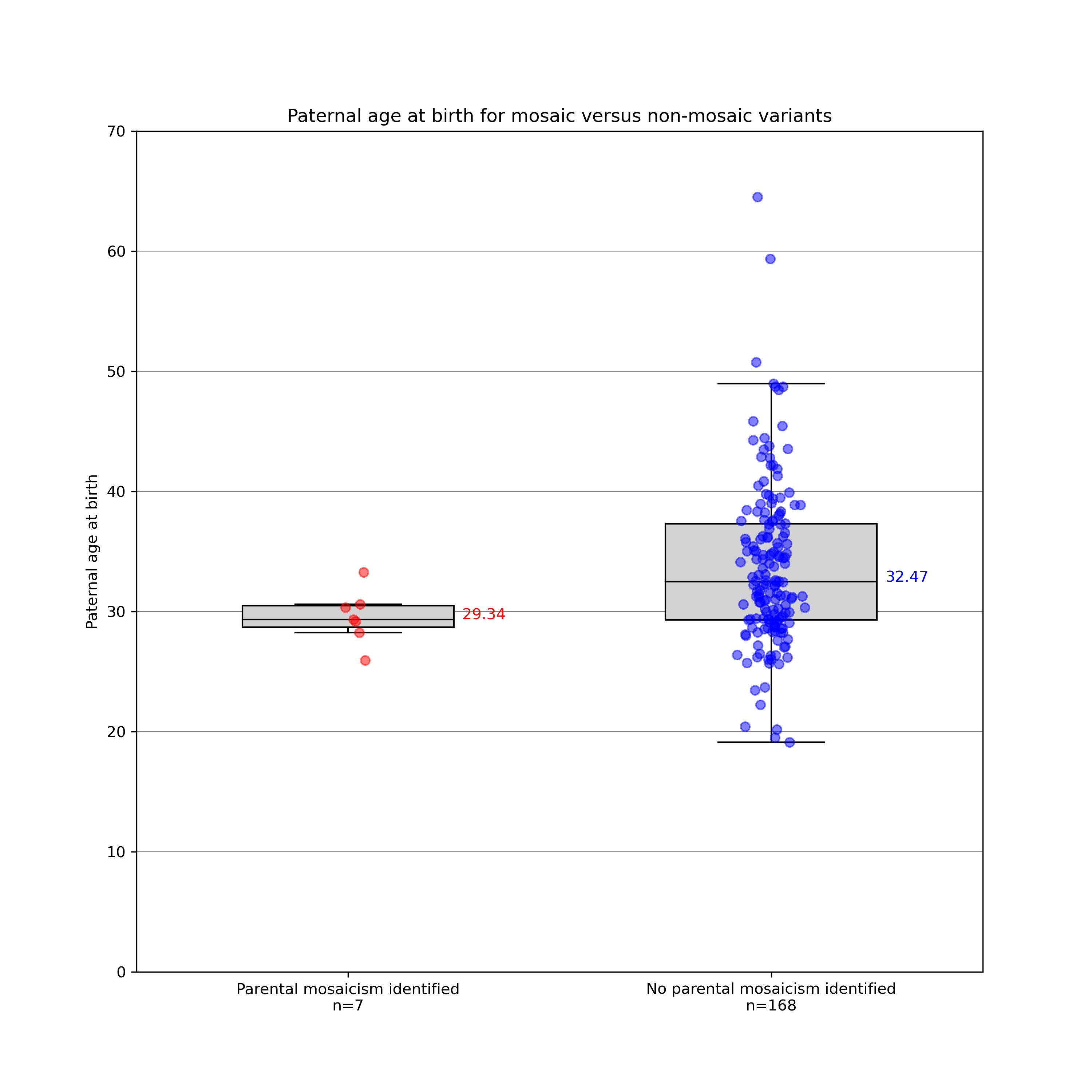


Supplementary Figure 4: Distribution of paternal age at birth according to mosaic status

Individual values of paternal age at conception are available in Table S1.
